# Conservation of Tubulin-Binding Sequences in TRPV1 throughout Evolution

**DOI:** 10.1371/journal.pone.0031448

**Published:** 2012-04-09

**Authors:** Puspendu Sardar, Abhishek Kumar, Anita Bhandari, Chandan Goswami

**Affiliations:** 1 National Institute of Science Education and Research, Bhubaneswar, Orissa, India; 2 Abteilung für Botanische Genetik und Molekularbiologie, Botanisches Institut und Botanischer Garten, Christian-Albrechts-Universität, Kiel, Germany; 3 Lehrstuhl für Molekulare Phytopathologie, Christian-Albrechts-Universität, Kiel, Germany; Duke University, United States of America

## Abstract

**Background:**

Transient Receptor Potential Vanilloid sub type 1 (TRPV1), commonly known as capsaicin receptor can detect multiple stimuli ranging from noxious compounds, low pH, temperature as well as electromagnetic wave at different ranges. In addition, this receptor is involved in multiple physiological and sensory processes. Therefore, functions of TRPV1 have direct influences on adaptation and further evolution also. Availability of various eukaryotic genomic sequences in public domain facilitates us in studying the molecular evolution of TRPV1 protein and the respective conservation of certain domains, motifs and interacting regions that are functionally important.

**Methodology and Principal Findings:**

Using statistical and bioinformatics tools, our analysis reveals that TRPV1 has evolved about ∼420 million years ago (MYA). Our analysis reveals that specific regions, domains and motifs of TRPV1 has gone through different selection pressure and thus have different levels of conservation. We found that among all, TRP box is the most conserved and thus have functional significance. Our results also indicate that the tubulin binding sequences (TBS) have evolutionary significance as these stretch sequences are more conserved than many other essential regions of TRPV1. The overall distribution of positively charged residues within the TBS motifs is conserved throughout evolution. *In silico* analysis reveals that the TBS-1 and TBS-2 of TRPV1 can form helical structures and may play important role in TRPV1 function.

**Conclusions and Significance:**

Our analysis identifies the regions of TRPV1, which are important for structure – function relationship. This analysis indicates that tubulin binding sequence-1 (TBS-1) near the TRP-box forms a potential helix and the tubulin interactions with TRPV1 via TBS-1 have evolutionary significance. This interaction may be required for the proper channel function and regulation and may also have significance in the context of Taxol®-induced neuropathy.

## Introduction

Transient receptor potential Vanilloid sub type 1, known as TRPV1 is a non-selective cation channel and the founder member of the TRPV subfamily. So far, TRPV1 gene has been sequenced from different species and endogenous expression of TRPV1 has been detected in several tissues and cells of certain species [1]. TRPV1 function has been linked with several sensory and other physiological functions that can certainly provide advantages for adaptation. For example, TRPV1 can detect noxious exogenous chemical stimuli at low concentration and thus warns the individual against potential damage to the system. For example, TRPV1 in most mammals can be activated by capsaicin (at micro molar or less concentration), the active pungent compound present in the hot chili. Interestingly, TRPV1 sequences in birds retains a single point mutation at the capsaicin binding site that results in total capsaicin insensitivity of TRPV1 [2]–[5]. This mutation is advantageous as birds can use chili as a food source. In return, this is also advantageous for chili plants as seeds can be dispersed at longer distance effectively by birds. There are other factors too, such as rate of seed germination, and resistant against fungal and bacterial infections that contributes to this co-evolution [6]. In addition, TRPV1 (in most species that are tested so far) can also be activated by low pH, another factor that can provide advantage for adaptation purpose [7]. Similarly, TRPV1 in most species is involved in the detection of temperature and thus seems to be important to distinguish favorable temperature from noxious temperature [8]–[9]. In bat, TRPV1 and a splice variant of TRPV1 is involved in the detection of infrared [10]. This property is certainly advantageous as bat use this property for navigation and control of locomotion. Similarly, in snake, TRPV1 is involved in detection of infrared and that allows them to identify pray successfully [11].

In addition, TRPV1 provides an excellent example of a polymodal receptor that can be activated simultaneously and synergistically by various physical and chemical stimuli [12]. As living habitats and environments encountered by different species is tightly linked with detection of noxious stimuli and further recruitment of downstream signaling, TRPV1 provides an example of a unique molecule that can be studied in the context of molecular evolution. The subtle changes and retention of domains and motifs that are essential for the function can also be explored by comparing the multiple sequences from different organisms. At the structural level, TRPV1 has six transmembrane (TM) regions and a pore-loop region. Both the N- and C-terminal cytoplasmic domains are located at the cytoplasmic side and are known to interact with several cytoplasmic proteins and other components. Interestingly, many of these interactions are important for proper function and regulation of these channels. Notably, the binding sites of these interacting proteins and components have been defined recently. For example, tubulin-binding sites, calmodulin binding sites, cholesterol binding motif, tetramer assembly domain and others within TRPV1 have been determined [13]–[15]. However, it is not known if these interactions and the functional regions are conserved throughout the evolution and have any importance in the context of structure – function relationship. In this work, we have retrieved the TRPV1 sequences from different species available in database and analyzed the molecular evolution of TRPV1. We specifically analyzed the domains, motifs and interacting regions that are conserved across these species and also analyzed the respective selection pressure imposed on these regions from their degree of conservation. This understanding uncovers hidden features in the TRPV1 sequences, especially in the tubulin-binding sequences that may be central to the structure – function relationship of TRPV1.

## Results

### TRPV1 protein has evolved during Silurian era

**Figure 1:**
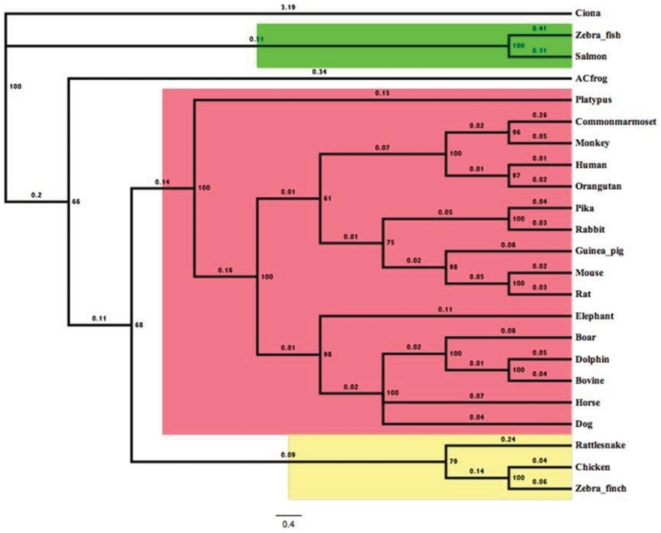
Bayesian phylogeny of TRPV1 illustrates that there is a single copy of this gene is conserved across different vertebrates. Bayesian phylogenetic tree of TRPV1 proteins from mammals (red) birds-reptiles (yellow) and fishes (green) was generated using MrBayes 3.2 [52]. Percentage posterior probabilities are marked at the node of the branches while mean branch length is marked in decimal on the respective branch. Putative TRPV like gene (JGI accession id e_gw1.02q.75.1) from “Ciona intestinalis” served as out-group in this phylogenetic tree.

**Figure 2:**
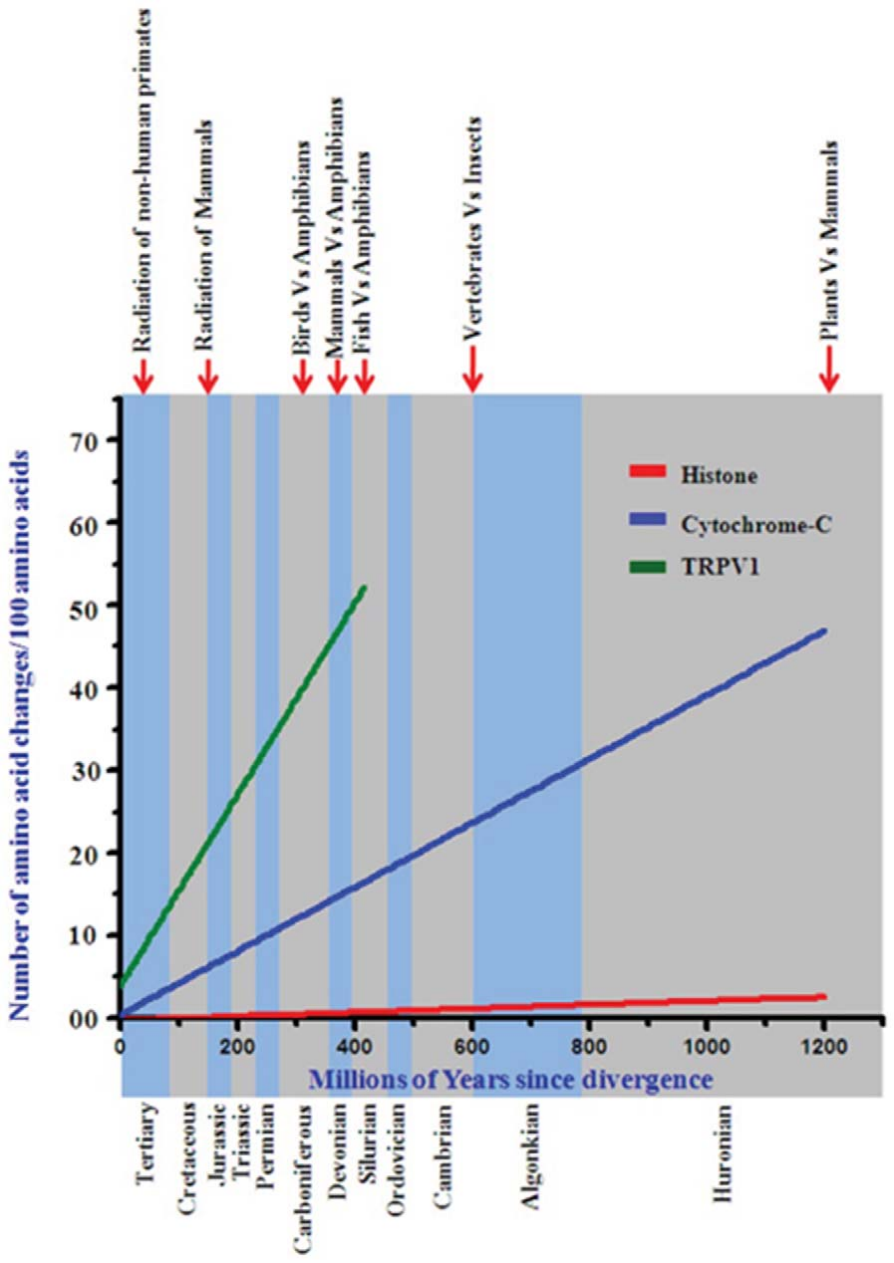
Molecular evolution of TRPV1. Conservation analysis with TRPV1 in comparison to histone H4 and cytochrome C.

We explored how TRPV1 protein has evolved during molecular evolution. For that purpose we have retrieved full-length or partial sequences of TRPV1 from available databank. Full-length protein sequences were used for the establishment of bayesian phylogenetic history of TRPV1, which depicts that there is a single copy of TRPV1 gene conserved across different lineages of vertebrates with high statistical support (**Figure 1**). This suggests that TRPV1 originated at point of emergence of vertebrates between 400 to 450 MYA, mostly during the transition of Silurian era from Devonian era. Furthermore, we calculated the evolutionary age and pattern of TRPV1. For that purpose we estimated the number of amino acid changes per 100 sites among different TRPV1 sequences [16] (**Figure 2**). We compared the conservation of full length TRPV1 channels in different species ranging from fish to human. For this analysis, we used histone-4 as an example of a highly conserved protein and cytochrome-C as a semi-conserved protein [16]–[17]. This analysis indicates that TRPV1 molecule has evolved during carboniferous era (approximately 400 MYA) before when amphibians developed from fishes.

### Different regions of TRPV1 have evolved through different selection pressure

**Figure 3:**
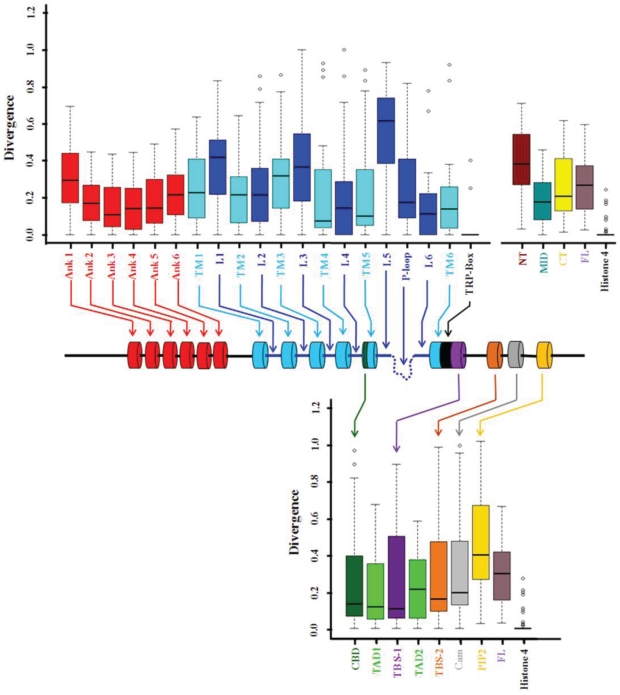
Analysis of conservation of different domains, motifs and interacting sites in TRPV1. The lower value indicates more conservation and higher value indicates less conservation. Different regions of the TRPV1 are indicated by different colors. Ank: Ankyrin repeat region, TM: Transmembrane region, L: Loop region, CBD: Cholesterol-binding domain, P-loop; Pore loop, TRP-box: Signature motif for TRP box, TBS-1: Tubulin binding sequence 1, TBS-2: Tubulin binding sequence 2, TAD-1: Tetrameric assembly domain 1, TAD-2: Tetrameric assembly domain 2, Cam: Calmodulin binding region, NT: N-terminal cytoplasmic domain of TRPV1, MID: Middle portion of TRPV1, CT: C-terminus of TRPV1, FL: Full length TRPV1, Histone 4: Histone 4. All values are significant (P<0.0001, Kuskal-Wallis test).

The human TRPV1 has 839 amino acids that comprise several signature motifs, 6 TM domains, a pore domain and the cytoplasmic domains [8]. We tested if the entire polypeptide of TRPV1 is conserved to a same extent throughout the evolution. To address that question, we have used statistical approach. Our analysis revealed that pooled sequences (from total 22 different species) of TRPV1 are conserved throughout evolution (p value≤0.0001) (**Figure 3**). Further, we have tested the conservation of different domains, motifs and functional regions present in TRPV1. For that purpose, we have separately compared the degree of conservation of these different regions. This analysis reveals that all the regions of TRPV1 that are functionally and structurally important are not conserved to the same extent and have a large degree of variance (**Figure 3**). Among all, the TRP-box motif present in the C-terminal cytoplasmic domain is the most conserved in all the species suggesting that the TRP-box in indeed is an authentic signature motif of TRPV1. This analysis also indicates the importance of other specific motifs. For example the cholesterol-binding CRAC motif present in the fifth TM domain of TRPV1 is less conserved than the fifth TM domain itself [15]. This probably suggests that cholesterol-mediated regulation of TRPV1 is a relatively new phenomena observed only in higher animals (discussed later). Similarly, among all TM domains, the fourth and fifth transmembrane regions are more conserved (more precisely bhasfourth TM). The sixth transmembrane is less conserved than the fifth TM. The third TM is the least conserved indicating that fourth and fifth TM regions are more important for the functional purpose. Similarly, among all ankyrin repeat domains, the first ankyrin repeat is less conserved. Interestingly, we found that loop sequence sixth, which connect the pore loop to the sixth TM region is highly conserved suggesting that the flexibility of this loop portion is important for the structure function relationship of TRPV1. In a reverse manner, fifth loop which links fifth TM region to the pore loop is the most divergent indicating that this portion of the loop is important but may not have tight sequence specificity for its function. Similarly, we noted that tubulin-binding stretch sequences are highly conserved. Interestingly, among these two, the TBS-1 is more conserved than TBS-2 suggesting that TBS-1 is important for some functional and regulatory purpose which seems to be conserved in all species (discussed later).

### TBS-1 and TBS-2 of TRPV1 form potential alpha helices containing positive charges in one side

**Figure 4:**
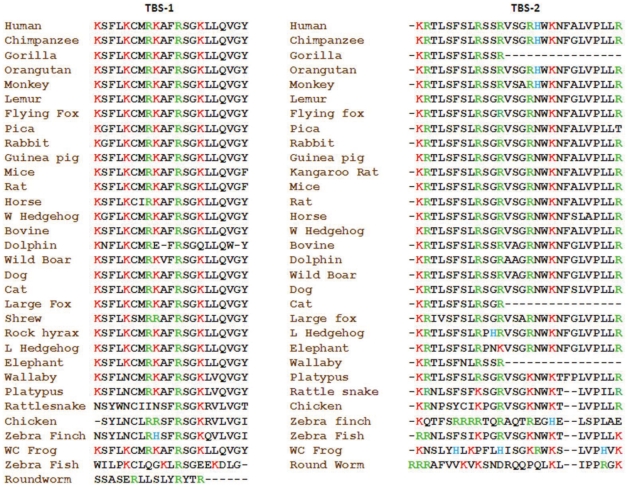
The primary sequence of TBS-1 and TBS-2 are semi-conserved throughout the evolution. The conservation of the basic amino acids is indicated.

**Figure 5:**
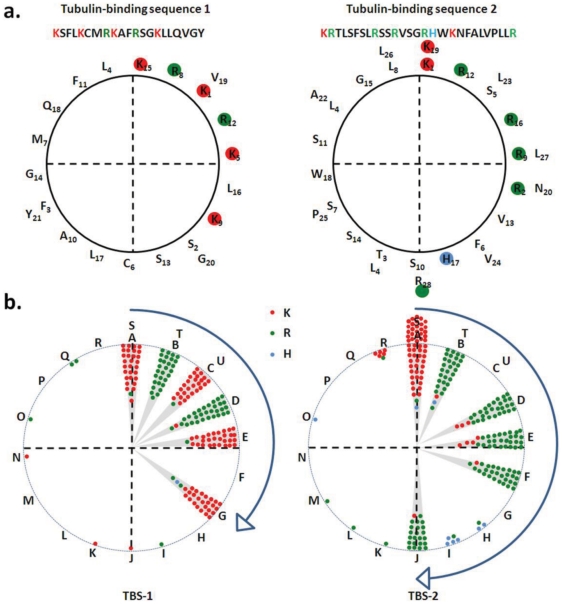
Distribution of positively charged residues within the TBS-1 and TBS-2 are evolutionary conserved. **a.** The distribution of positively charged residues located within TBS-1 and TBS-2 of human TRPV1 are represented in a 360° circular wheel. **b.** The helix formed by TBS-1 and TBS-2 from different species are superimposed and the distribution of positively charged residues are represented in a 360° circular wheel. The positions are marked by alphabets and A indicated the position of first positively charged residues in the helical wheel that has been placed at 0° angle. The distribution of positively charged residues in both TBS-1 and TBS-2 remain much conserved. Residues - lysine (K), arginine (R) and histidine (H) are represented by red green, and the indigo dots, respectively.

Previously, we described that the TBS-1 and TBS-2 sequences are semi-conserved among mammals [13]. We also suggested that TBS-1 and TBS-2 may form alpha helices as all the positively charged residues are distributed in one side if plotted as helices [13], [18]. Thus we explored if TBS-1 and TBS-2 from all other species can also adapt similar helical structure and if such helical structure has any evolutionary significance. For that purpose, we have aligned the TBS-1 and TBS-2 sequences and have noted that these sequences are semi-conserved (**Figure 4**). We noted that though the overall sequence may change, the distribution of positively charged residues remain highly conserved in most of the species, corroborating the importance of these residues. We have plotted the TBS-1 and TBS-2 sequence from different species as alpha helices. We noted that the distribution of positively charged residues occurs mostly in one side of the helices (**Figure 5a**). When all these potential helices from different species were merged together, the conserved distribution of positively charged residues to one side of the helices becomes prominent (**Figure 5b**). This analysis also indicate that TBS-1 actually represent a “+XXX+XX++XX+XX+XXXXXX” motif sequence where+can be any positively charged residues (mostly lysine or arginine) and X can be any amino acid except negatively charged residues and helix breaking residues. These results strongly suggest that TBS-1 and TBS-2 may involve in the interaction with some poly-anionic stretch sequences and among which TBS-1 seem to play an important function that is not only critical for the TRPV1 ion channel function but also conserved though out the evolution.

## Discussion

In this study, we have analyzed the conservation of different domains and motifs and interacting sites. Such analysis is especially important as still now crystal structure of TRPV1 is not available and thus, the structural information about different domains and motifs still remain speculative. We have retrieved TRPV1 sequences from 34 different species and analyzed the molecular evolution of TRPV1, we found that a single copy of TRPV1 gene is conserved across vertebrates (**Figure 1**). We demonstrate that TRPV1 has evolved nearly 416 MYA. These analyses shade important information about the structure - function relationship of TRPV1 and a much more information can be interpreted from such analysis. For example, analysis of changes per 100 amino acids indicates that unlike histone and cytochrome-C (examples of highly conserved and semi-conserved proteins respectively), TRPV1 is neither a highly conserved protein nor has evolved very early in evolutionary history. Our analysis indicates that TRPV1 is an example of a protein that has not yet reached to its optimal structure – function relationship by molecular evolution. This is particularly due to the fact that line representing the conservation of TRPV1 does not intersect Y-axis at its origin (**Figure 2**). Therefore, considering the current rate of changes, it can be hypothesized that another ∼40 MYA is needed when TRPV1 will have its optimal structure – function relationship. However, the TRP-box has reached its optimum structure and function. This is due to the fact that the TRP-box motif is highly conserved in all species and nature has excluded any substitution in this region.

However, our analysis gives important information of different domains and motifs. For example, the analysis shades important information of the tubulin-binding sequences and their nature, probable function and structure. The positively charged residues in both TBS-1 and TBS-2 are located one side of the helix if TBS-1 and TBS-2 are plotted as alpha helices [13]. Previously we have reported that the two tubulin-binding sequences, namely TBS-1 and TBS-2 are present in TRPV1 have high isoelectric points (pI values 11.17 for TBS-1 and 12.6 for TBS-2 respectively, rat sequence) due to the presence of several positive charged residues [13]. In this work we also demonstrate that positively charged residues within TBS-1 and TBS-2 are highly conserved in different species (**Figure 4**). Among all species, the TBS-1 has an average pI of 10.52 (highest 11.74 and lowest value 8.18). In a similar manner, the TBS-2 reveals an average pI of 12.41 (highest 12.6 and lowest value 10.68). This conservation of positively charged residues and high pI of these two stretches strongly suggest that the positively charged residues are important for some functional aspects.

A significant understanding of the function and nature of the TBS-1 and TBS-2 can also be derived from the substitution of its amino acids. We noted that within a fixed position, though there are some substitutions of positively charged residues, these kinds of substitutions are much less in stretch 1 compared to stretch 2. In addition, in case of stretch 1, majority (95.98%) of the positively charged residues are located within 120° angle while in case of stretch 2, majority (95.35%) of the positively charged residues are located within 180° angle. This clustering of positively charged residues in one side of the helix suggests two aspects: first, these stretch sequences can indeed form alpha helices and this helical arrangement can facilitates interaction with proteins that contains negatively charged residues (like C-terminal region of tubulin). Indeed, predict protein software (freely available in https://www.predictprotein.org/ site) analysis reveals that TBS-1 (rat sequence) sequence contains 66.67% alpha helix and 33.33% loop. The high degree of conservation also suggests that the TBS-1 and TBS-2 (preferably the first one) have functional importance, which is conserved throughout the evolution. We propose that interaction with poly-anionic stretch sequences is one of such function. In this regard, it is important to mention that the C-terminal regions of all alpha- and beta-tubulin have E-hook sequences that contain multiple negatively charged residues and these E-hooks are intrinsically unstructured [19]–[20]. This hypothesis is supported by the fact that negatively charged residues are selectively excluded from both TBS-1 and TBS-2.

Selective exclusion of histidine, another basic amino acid from TBS-1 and especially in the key positions throughout the evolution is suggestive of the environment and function of this region. In this context, it is important to note that at pH 7.4, only 10% of the histidine (H) will have positive charge and 90% will be neutral. Therefore, exclusion of histidine from this region and lack of substitution of lysine (L) and arginine (R) by H in this TBS-1 indicate that positive charges in TBS-1 are required mostly in the neutral pH range. Therefore it can also be concluded that the TBS-1 is not exposed to a pH range that is lower than 7.4, a condition where histidine will provide positive charge. Interestingly, both of TBS-1 and TBS-2 are characterized by major absence of negatively charged residues (Only 0.685% E and 0.156% D in stretch 1 and 0.360% E and 0.115% D in stretch 2) in all the species for which the sequences are available (**Figure 4**). It is noteworthy that majority of TBS1 and TBS2 sequences do not possess either E or D. Out of 32 sequences available from different species, 30 sequences do not have any E or D in case of TBS-1. Similarly, in case of TBS-2, 29 sequences do not have any E or D out of 31 sequences.

To analyze if these two sequence stretches can indeed form alpha-helices, we used an indirect approach and analyzed the occurrence of helix-braking amino-acids in TBS-1 and TBS-2 two stretch sequences in all the species. We noted that within these stretch sequences, the occurrence of proline (P), a helix breaker is very less (0.156% in stretch 1) and only present in case of zebrafish sequence (only one P residue occurs within the stretch 1). In contrast, the occurrence of proline is bit high in TBS-2 (4.322%) and at least one P is conserved in all the sequences except wallaby, gorilla and cat (the sequences are not complete in these species). In contrast to the helix breaking amino acids, helix-forming amino acids (M, A, L, E and K) are selectively enriched within these stretches (3.779% alanine amino acid, 14.116% leucine, 3.884%M, 0.685%E, 16.281%K in TBS-1) and (0.00% M, 2.588% alanine amino acid, 16.637% leucine, 0.360%E, 8.507%K in TBS-2). These results strongly suggest that helix-braking and negatively charged amino-acids are selectively excluded and helix forming amino acids were included and/or retained during evolution, at least in case of TBS-1. As the TBS-1 is overlapping with TRP-box, a region that is involved in the channel function regulation, we suggest that TBS-1 and TBS-2 are also important for the channel function. Interaction of tubulin with TBS-1 may have importance for the channel function too and thus explain why this motif sequence remains conserved throughout the evolution. Considering all these facts, our analysis suggests that TBS-1 may form a putative helix projecting all the basic amino acids in one side where the E-hook sequence of tubulin interacts. Based on this understanding, we hypothesized that tubulin interaction is possible with other TRP channels that contain “+XXX+XX++XX+XX+XXXXXX” motif sequence. Indeed, we noted the presence of the critical features of this motif sequence in other TRP channels other than TRPV1, namely in TRPV2, TRPV3, TRPV4, TRPV5, TRPV6, TRPC1, TRPC2, TRPC3, TRPC4, TRPC5, TRPC6 and TRPM8 (date not shown).

Though at present the exact functional importance of this motif sequence is not clear, this motif represents a hidden signature preset in many TRP channels that is most likely of functional importance. With the advancement in sequencing of several genomes in last two decades, it is evident that various protein families or classes are specified by the presence of protein signatures and/or motifs that are of functional and evolutionary significance. There are several examples which support this view. For example, G-protein coupled receptors [21]–[23], serine proteases [24], serine protease inhibitors [25]–[26], sorting nexins [27] and the eight-cysteine motif in plants [28] contain motifs that are primarily considered to have physiological importance and are conserved across different species. In agreement with that, a recent study has compared the protein sequences of TRPA1 from different reptiles and identified 21 amino acids that are important for infrared detection in snakes that contain pit organs [29]. Similarly, in this work we report conservation of important protein motifs present in TRPV1, and suggest that these motifs play important functional roles that is yet to discover. In future we will explore these possibilities in detail with experimental evidences.

We suggest that the tubulin interaction with TRP channels has importance in the context of chemotherapy-induced neuropathy where administration of microtubule stabilizer based chemotherapeutics such as Taxol and Vinca drugs are known to induce neuropathic pain and other pathophysiological disorders [30]–[36]. One of the potential target of Taxol®-induced neuropathy can the TRP channels. Previously, alteration in the pain threshold due to application of Taxol® has been demonstrated [37]. In agreement with these observations, recently we have demonstrated that TRPV1 and TRPV4, commonly termed as pain receptors interact physically and functionally with microtubules as well as with the soluble tubulin dimers [13], [18], [38]–[44]. At the molecular level, how Taxol or other microtubule stabilizers affect TRPV channels that remain to explore.

## Materials and Methods

### Sequence retrieval and alignment

The TRPV1 sequences were retrieved from Ensemble [45]–[46] and National Centre for Biotechnology Information (NCBI) database [47]–[48]. The TRPV1 sequence from snake (*Crotalus atrox*) was retrieved from NCBI [11]. Details of each gene and protein are given in the figure section as tabular form (**table S1**). The sequence alignment was done by using MUSCLE alignment software [49]–[50] with its default values. As a highly conserved protein, sequences for histone H4 from different species were downloaded from the Ensembl site (http://www.ensembl.org/index.html) [45]–[46] (**table S2**). Similarly, sequences for cytochrome C (a semi-conserved protein) were also downloaded from different databases [16] (**table S3**).

### Phylogenetic tree formation

We used MUSCLE alignment program to align the amino acid sequences of TRPV1 for the purpose of phylogenetic analysis [49]–[50]. We implemented a Bayesian phylogenetic tree constructed by the Bayesian approach (5 runs, 7500,000 generations, 25% burn-in-period, WAG matrix-based model in the MrBayes 3.2 program [51]–[52].

### Fragmentation of TRPV1 in different domains and motifs

To analyze the conservation of different small regions of the TRPV1 that are important structurally and/or functionally, different domains and motifs characterized before were analyzed separately. These regions were selected as described previously (**Table 1**). For that purpose, different structural elements such as the N-terminal cytoplasmic domain [8], ankyrin repeats - 1–6 [53]–[54], all transmembrane regions (TM1–6), all six loops, pore domain and the C-terminal cytoplasmic domain were analyzed [8]. The tubulin-binding regions present in the C-terminal region of TRPV1 were selected as mentioned before [13]. To find out the Calmodulin-binding site on TRPV1, full length Rat TRPV1 was used as quarry sequences and submitted to the UniProt (http://www.uniprot.org, accession id O35433-2). This matches well with the previous report describing the physical binding site of calmodulin on the C-terminal region of TRPV1 [55]. The position of TRP-box and PIP2 binding site was followed as described previously [56]–[57]. The tetramerization assembly domains (TAD1 and TAD2) located within the C-terminal region of TRPV1 was followed as described before [58]–[59]. The cholesterol-binding region, namely the CRAC motif located within the fifth TM region has been considered as described recently [15].

**Table 1:** Description of different domains and motifs.

Region	Location (Amino acid number)	References
N-terminal	1–432	[8]
C-Terminal	684–838	[8]
Ank-1	112–152	[53]
Ank-2	153–199	[53]
Ank-3	200–246	[53]
Ank-4	247–282	[53]
Ank-5	283–331	[53]
Ank-6	332–359	[53]
TM-1	433–454	[8]
Loop-1	455–479	[8]
TM-2	480–495	[8]
Loop-2	496–509	[8]
TM-3	510–531	[8]
Loop-3	532–542	[8]
TM-4	543–569	[8]
Loop-4	570–576	[8]
Cholesterol binding domain	577–593	[15]
TM-5	577–596	[8]
Loop-5	597–623	[8]
Pore region	624–645	[8]
Loop-6	646–654	[8]
TM-6	655–683	[8]
TAD-1	684–721	[58]
TRP-Box	696–701	[58]
TBS-1	710–730	[13]
TAD-2	752–772	[59]
TBS-2	770–797	[13]
Cam-BR	767–801	[14]
PIP-2	778–819	[57]

In all cases, the rat TRPV1 sequence (Ensembl accession id ENSRNOP00000026493) was used as the template. Specific domain and motif sequences described for other species were used as quarry in order to find the corresponding regions present in the rat TRPV1 and also in TRPV1 sequences from different species. MUSCLE software was used to align and find out the respective regions present in other species. The aligned data were subsequently imported into R statistical tool for statistical analysis. As the complete TRPV1 sequences from certain species are not available (mostly due to sequencing errors at certain regions), the analysis aimed to understand the conservation of different domains and motifs of TRPV1 were conducted with the available sequences only (**table S1**). We omitted using these incomplete sequences in cases where full-length sequences are needed.

### Distance Matrix generation and Statistical tests

Using the saved alignment files in MEGA5 distance matrices were generated for different aligned data sets [60]. Using this method, pair-wise distances of any two different amino acid sequences within a group can be measured. To estimate the variance, bootstrap method was used. In substitution method, amino acid p-distance was used. In case of data gaps/data missing pair-wise deletion method was used. For each data set there will be one matrix which informs about the pair-wise distances of all sequences in a group. In the matrix window distances between each sequence with another is calculated along with overall mean distance of all sequences.

Then the pair-wise distance values (generated in the distance-matrix) were imported in “R” software for statistical analysis and graphical representation. Using R, box-plots were generated to represent the evolutionary relationship of different protein sequences. The Kruskal-Wallis analysis of variance test was done for each set of data to check the reliability and significance of the data points [61]. As we have measured the pair-wise evolutionary distances of protein sequences, the graphical representation reflect values in the Y-axis which is inversely proportional with the conservation. Therefore, the conserved sequences show lower values and divergent sequences show higher values in the Y-axis. Along with this calculation, the median values of each data set were calculated and also represented along with conservation figures.

### Helical wheel plotting

The tubulin-binding sequences for both stretch 1 and stretch 2 in different species were plotted as helices by using pepwheel online software (available at http://emboss.bioinformatics.nl/cgi-bin/emboss/pepwheel). Steps 18 and Turn 7 parameters were set in the software to plot the wheel. After plotting the sequences as helical wheels, the images were saved as .png format for further analysis. Distributions of positive charged amino acids present in all species were manually plotted on two different circles (representing TBS-1 and TBS-2) by superimposing all the images. Specific zones of positive charged amino acids were marked manually on these circles. The circles were further divided in four quadrants as described in the **figure 5**. The regions where all the positively charged residues are clustered within these two circles are marked with gray background.

### Calculation of evolutionary time

The presence of TRPV channels is restricted to higher eukaryotes only. The lowest organism where, any TRP channel is documented so far is yeast [62]–[63]. In order to explore the molecular evolution of TRPV1 in the term of million years, we compare the sequences among different classes and number of changes of amino acids per 100 amino acids was calculated by comparing birds with reptiles, fish with reptiles and reptiles with mammals, etc for all available TRPV sequences [16]. The Human TRPV sequences are taken as the most recent one in the history of evolution. Therefore, the evolutionary time of Human TRPV sequences is considered as zero (0) million year. We calculated average change and we plotted radiations of mammalian TRPV sequences against million years.

While calculating radiation of non-human primates for TRPV1 sequence then monkey (ENSMMUP00000000966) TRPV1 was compared with human (ENSP00000174621) TRPV1 sequence, and while calculating total mammalian radiation then mouse (ENSMUSP00000099585), bovine (ENSBTAP00000025131), dog (ENSCAFP00000028570), horse (ENSECAP00000020491), elephant (ENSLAFP00000011058), pika (ENSOPRP00000015398) and boar (ENSSSCP00000018922) TRPV1 sequences were compared with human (ENSP00000174621) TRPV1 sequence separately and average amino acid change/100 amino acids was calculated. For calculation of birds with amphibian, chicken (ENSGALP00000007393) and Zebra Finch (XP_002195940.1) TRPV1 sequences were compared with *Xenopus* (NP_001177322.1) TRPV1 sequence separately and average amino acid change/100 amino acids ware calculated. In the similar way amino acid change/100 amino acids was calculated for fish with amphibian by comparing *Xenopus* (NP_001177322.1) against zebrafish (ENSDARP00000078166) and salmon fish (GenBank ID: ACI34236). Comparison between amphibians with mammals was performed by comparing human TRPV1 sequence with *Xenopus* TRPV1.

## Supporting Information

Table S1 **Source, accession numbers and corresponding databases of TRPV1 sequences from different species.** The lengths of the amino acids for which the sequence are available are indicated.
(DOCX)

Table S2 **Accession ID, isoforms, transcript IDs and corresponding lengths of Histone sequences from different species.**
(XLSX)

Table S3 **Protein ID and length of Cytochrome C from different species.**
(DOCX)
