# Supplementary material for: Conservation of Tubulin-Binding Sequences in TRPV1 throughout Evolution
Source: PLoS One. 2012 Apr 9;7(4):e31448. doi: 10.1371/journal.pone.0031448 (PMC3322131; doi:10.1371/journal.pone.0031448)
Supplement: Table S1 — Source, accession numbers and corresponding databases of TRPV1 sequences from different species. The lengths of the amino acids for which the sequence are available are indicated. (DOCX) [file pone.0031448.s001.docx]

**Supplementary table 1**

| **Organisms** | ***Scientific Name*** | **Gene ID** | **Protein ID** | **Length (aa)** |
| --- | --- | --- | --- | --- |
| **Full length sequences** |  |  |  |  |
| **From Ensembl** |  |  |  |  |
| Human | *Homo sapiens* | **ENSG00000196689** | **ENSP00000174621** | 839 |
| Mouse | *Mus musculus* | ENSMUSG00000005952 | ENSMUSP00000099585 | 839 |
| Rat | *Rattus norvegicus* | ENSRNOG00000019486 | ENSRNOP00000026493 | 838 |
| Bovine | *Bos Taurus* | ENSBTAG00000018880 | ENSBTAP00000025131 | 837 |
| Common Marmoset | *Callithrix jacchus* | ENSCJAG00000007063 | ENSCJAP00000013185 | 842 |
| Dog | *Canis familiaris* | ENSCAFG00000019339 | ENSCAFP00000028568 | 850 |
| Guinea pig | *Cavia porcellus* | ENSCPOG00000010142 | ENSCPOP00000009106 | 839 |
| Horse | *Equus caballus* | ENSECAG00000022758 | ENSECAP00000020491 | 837 |
| African Bush Elephant | *Loxodonta africana* | ENSLAFG00000013211 | ENSLAFP00000011058 | 840 |
| Rhesus Macaque | *Macaca mulatta* | ENSMMUG00000000711 | ENSMMUP00000000966 | 838 |
| American Pika | *Ochotona princeps* | ENSOPRG00000016865 | ENSOPRP00000015398 | 843 |
| Platypus | *Ornithorhynchus anatinus* | ENSOANG00000012698 | ENSOANP00000020088 | 849 |
| European Rabbit | *Oryctolagus cuniculus* | ENSOCUG00000012228 | ENSOCUP00000010529 | 853 |
| Orangutan | *Pongo pygmaeus* | ENSPPYG00000007815 | ENSPPYP00000008801 | 837 |
| Wild Boar | *Sus scrofa* | ENSSSCG00000017863 | ENSSSCP00000018922 | 827 |
| Bottlenose dolphin | *Tursiops truncates* | ENSTTRG00000001203 | ENSTTRP00000001128 | 843 |
| Chicken | *Gallus gallus* | ENSGALG00000004649 | ENSGALP00000007393 | 843 |
| Zebrafish | *Danio rerio* | ENSDARG00000059883 | ENSDARP00000078166 | 819 |
| **From NCBI** |  |  |  |  |
| Zebra finch | *Taeniopygia guttata* | XP_002195940.1 |  | 844 |
| Affrican clawed frog | *Xenopus laevis* | NP_001177322.1 |  | 838 |
| Salmon | *Salmo salar* | GenBank: ACI34236 |  | 804 |
| Rattle snake | *Crotalus atrox* | GeneBank ID: GU562968 |  |  |
| **Not fully sequenced** |  |  |  |  |
| Cat | *Felis catus* | ENSFCAG00000007921 | ENSFCAP00000007337 | 784 |
| Gorilla | *Gorilla gorilla* | ENSGGOG00000009871 | ENSGGOP00000009652 | 525 |
| Kangaroo rat | *Dipodomys ordii* | ENSDORG00000009857 | ENSDORP00000009265 | 755 |
| Lesser hedgehog | *Echinops telfairi* | ENSETEG00000007781 | ENSETEP00000006322 | 640 |
| West European Hedgehog | *Erinaceus europaeus* | ENSEEUG00000013355 | ENSEEUP00000012186 | 788 |
| Flying Fox | *Tarsius syrichta* | ENSTSYG00000006457 | ENSTSYP00000005912 | 711 |
| Large Flying Fox | *Pteropus vampyrus* | ENSPVAG00000012735 | ENSPVAP00000012011 | 804 |
| Gray Mouse Lemur | *Microcebus murinus* | ENSMICG00000015702 | ENSMICP00000014312 | 585 |
| Shrew | *Sorex araneus* | ENSSARG00000004344 | ENSSARP00000003962 | 434 |
| Rock hyrax | *Procavia capensis* | ENSPCAG00000011898 | ENSPCAP00000011140 | 617 |
| Wallaby | *Macropus eugenii* | ENSMEUG00000014774 | ENSMEUP00000013491 | 673 |
| Western clawed frog | *Xenopus tropicalis* | ENSXETG00000005790 | ENSXETP00000012743 | 690 |
