# Supplementary material for: Conservation of Tubulin-Binding Sequences in TRPV1 throughout Evolution
Source: PLoS One. 2012 Apr 9;7(4):e31448. doi: 10.1371/journal.pone.0031448 (PMC3322131; doi:10.1371/journal.pone.0031448)
Supplement: Table S3 — Protein ID and length of Cytochrome C from different species. (DOCX) [file pone.0031448.s003.docx]

**Supplementary table 3 (Cytochrome C)**

| **Organisms** | ***Scientific Name*** | **Protein ID** | **Source** | **Length (aa)** |
| --- | --- | --- | --- | --- |
| Human | *Homo sapiens* | NP_061820.1 | NCBI Reference Sequence | 105 |
| Pig | *Sus scrofa* | NP_001123442.1 | NCBI Reference Sequence | 105 |
| Zebrafish | *Danio rerio* | NP_001002068.1 | NCBI Reference Sequence | 104 |
| Drosophila | *Drosophila melanogaster* | AAF53553.1 | GenBank | 105 |
| Horse | *Equus caballus* | NP_001157486.1 | NCBI Reference Sequence | 105 |
| Bovine | *Bos Taurus* | NP_001039526.1 | NCBI Reference Sequence | 105 |
| Chicken | *Gallus gallus* | NP_001072946.1 | NCBI Reference Sequence | 105 |
| Dog | *Canis familiaris* | NP_001183974.1 | NCBI Reference Sequence | 105 |
| Chimpanzee | *Pan troglodytes* | NP_001065289.1 | NCBI Reference Sequence | 105 |
| Rat | *Rattus norvegicus* | AAA21711.1 | GenBank | 105 |
| Zebra finch | *Taeniopygia guttata* | NP_001137145.1 | NCBI Reference Sequence | 105 |
| Orangutan | *Pongo pygmaeus* | NP_001124639.1 | NCBI Reference Sequence | 105 |
| Mouse | *Mus musculus* | CAA25899.1 | GenBank | 105 |
| Honeybee | *Apis mellifera* | NP_001170961.1 | NCBI Reference Sequence | 108 |
| Salmon | *Salmo salar* | ACM08959.1 | GenBank | 104 |
| Lizard | *Varanus varius* | P21665.2 | Swiss-Prot | 105 |
| Arabidopsis | *Arabidopsis thaliana* | AAB72175.1 | GenBank | 114 |
| Rice | *Oryza sativa* (Indica Group) | AAA63515.1 | GenBank | 112 |
